# Association between Blood 25-Hydroxyvitamin D Levels and Survival in Colorectal Cancer Patients: An Updated Systematic Review and Meta-Analysis

**DOI:** 10.3390/nu10070896

**Published:** 2018-07-13

**Authors:** Haifa Maalmi, Viola Walter, Lina Jansen, Daniel Boakye, Ben Schöttker, Michael Hoffmeister, Hermann Brenner

**Affiliations:** 1 Division of Clinical Epidemiology and Aging Research, German Cancer Research Center (DKFZ), Im Neuenheimer Feld 581, Heidelberg 69120, Germany; h.maalmi@DKFZ.de (H.M.); viola.walter@gmx.net (V.W.); l.jansen@DKFZ.de (L.J.); d.boakye@DKFZ.de (D.B.); b.schoettker@DKFZ.de (B.S.); m.hoffmeister@DKFZ.de (M.H.); 2 Network Aging Research (NAR), Heidelberg University, Bergheimer Strasse 20, Heidelberg 69115, Germany; 3 German Cancer Consortium, German Cancer Research Center (DKFZ), Im Neuenheimer Feld 280, Heidelberg 69120, Germany

**Keywords:** vitamin D, mortality, colorectal cancer, cohort studies, meta-analysis, dose-response

## Abstract

Previous meta-analyses have shown an improved survival with higher blood 25-hydroxyvitamin D (25(OH)D) concentrations in patients with colorectal cancer (CRC). However, a number of much larger studies have been published since then. We provide an updated meta-analysis to synthesize current evidence. PubMed and Web of Science databases were systematically searched for eligible studies. The dose-response relationships and pooled hazard ratios for overall and CRC-specific survival comparing the highest versus the lowest categories of blood 25(OH)D concentrations were assessed. Subgroup analyses based on study geographic location, year of publication, sample size, length of follow-up time and stage were conducted to explore potential sources of heterogeneity. Overall, 11 original studies with a total of 7718 CRC patients were included. The dose-response meta-analysis showed an improvement in survival outcomes with increasing blood 25(OH)D concentrations. Pooled hazard ratios (95% confidence intervals) comparing highest versus lowest categories were 0.68 (0.55–0.85) and 0.67 (0.57–0.78) for overall and CRC-specific survival, respectively. Associations were more prominent among studies conducted in Europe, with larger sample sizes, and including stage I–IV patients. This updated meta-analysis reveals robust evidence of an association between higher blood 25(OH)D concentrations and better survival in CRC patients. The potential for enhancing prognosis of CRC patients by vitamin D supplementation should be explored by randomized trials.

## 1. Introduction

Colorectal cancer (CRC) is the fourth most common cause of cancer-related deaths globally, with more than 1.1 million cancer deaths expected by 2030 [1]. The identification of modifiable prognostic factors is highly desirable to improve the management of CRC patients and prognosis.

The role of vitamin D in CRC has been a topic of considerable interest. This interest started more than 30 years ago when the first ecological study reported an inverse association between solar radiation and CRC mortality [2]. Researchers attributed this association to the quantity of vitamin D synthesized in the skin after exposure to ultraviolet-B (UVB) radiation. Most prospective observational studies that have investigated an association with prognosis in CRC patients found higher blood 25-hydroxyvitamin D (25(OH)D) concentrations, the best indicator of vitamin D status in the body, to be associated with better survival [3,4,5].

To date, a number of reviews [6,7,8], meta-analyses [9,10,11] and dose-response meta-analyses [12,13] have summarized the results of five cohort studies that investigated the association between blood 25(OH)D concentrations and survival in patients with CRC. However, due to the small numbers of included studies and patients, none of the previous reviews could explore the potential variation of this association according to a specific study or patient characteristics. A number of much larger cohort studies have been published since which may strongly improve the statistical power, increase the precision of pooled estimates and help to evaluate the association within subgroups of studies or patients.

Therefore, we conducted an updated systematic review and meta-analysis to comprehensively evaluate the relationship between blood 25(OH)D levels and survival, with a particular focus on associations within subgroups and pooled dose-response relations.

## 2. Materials and Methods 

### 2.1. Search Strategy

The reporting of meta-analyses of observational studies in epidemiology (MOOSE) guidelines were followed to perform this systematic review and meta-analysis [14] (Table S1). We carried out a systematic literature search in PubMed and Web of Science databases for articles reporting results of cohort studies conducted in CRC patients and assessing the association between blood 25(OH)D concentrations and overall and CRC-specific survival, using a comprehensive list of search terms (Table S2). The current literature search was restricted to articles published from 2013 until September 2017 with no language restrictions, thereby complementing our previous corresponding literature search of articles published up to 2013 [10].

### 2.2. Selection

We excluded studies (i) with non-longitudinal design; (ii) restricted to non-CRC patients; (iii) without measurement of blood 25(OH)D and (iv) without measurement of the association between exposure and outcome of interest. In addition, we excluded conference abstracts due to the insufficient data obtainable from them.

### 2.3. Data Extraction

From each included study, data were independently extracted by two investigators (H.M and V.W) using a standardized data extraction form. Briefly, we recorded study characteristics including first author name, year of publication, a country in which study was conducted, study acronym and time period of study conduction (period of recruitment and mean/median duration of follow-up). We also extracted the following information about study populations: sample size, sex, CRC stage, age and number of total and CRC deaths. In addition, we extracted data about the time between 25(OH)D measurement and cancer diagnosis, categories of blood 25(OH)D concentrations and the median/mid-point/interval of 25(OH)D concentrations in each category. Finally, we recorded hazard ratios (HRs) and 95% confidence intervals (CIs) for the association of 25(OH)D with overall and CRC-specific survival as well as confounders adjusted for in the analysis. Disagreements between investigators were discussed and resolved by an additional review. In this report, blood 25(OH)D concentrations were expressed in nmol/L. To convert concentrations reported in ng/mL, an adequate conversion factor (1 ng/mL = 2.5 nmol/L) was used.

### 2.4. Statistical Analysis 

#### 2.4.1. Meta-Analysis

The pooled hazard ratios (HR) and 95% confidence intervals (CI) for the association of categories of patients with highest vs. lowest 25(OH)D concentrations with overall and CRC-specific survival were estimated using the DerSimonian and Laird (DL) random-effects model to account for heterogeneity of study populations and designs [15]. The heterogeneity among the included studies was investigated using the I^2^ index and Cochran’s Q test, with significant heterogeneity assumed for I^2^ > 50% or a *Q*-test *p*-value < 0.05 [16]. Subgroup analyses were conducted to explore potential sources of heterogeneity across studies. Publication bias was assessed graphically with funnel plots and statistically with Kendall’s tau [17] and Egger’s test [18]. A *p*-value < 0.05 in these tests suggests the presence of publication bias. Sensitivity analyses were performed, to test the stability of the pooled HR estimates and 95% CIs, by exclusion/or inclusion of some specific studies. Study quality was assessed based on the adjustment level used in each included study, considering age, sex and season which are among the most important potential confounders. CRC-specific survival was not reported in many studies. Therefore, sensitivity and subgroup analyses were only performed for overall survival. For these analyses, we used the R statistical software, version 3.3.2, and package “metaphor”, version 2.0 (R Foundation for Statistical Computing, Vienna, Austria).

#### 2.4.2. Dose-Response Meta-Analysis 

To perform dose-response analyses, information on blood 25(OH)D concentrations were extracted from each study directly if the concentration for each 25(OH)D category was reported as either mean, median, or mid-point. If 25(OH)D concentrations were reported only as intervals, mid-points were calculated. For each individual study, we plotted HRs and their 95% CIs according to 25(OH)D concentrations in order to compare the dose-response pattern across studies. For this analysis, studies having a total number of deaths <400, were excluded.

## 3. Results

### 3.1. Search Results

**Figure 1:**
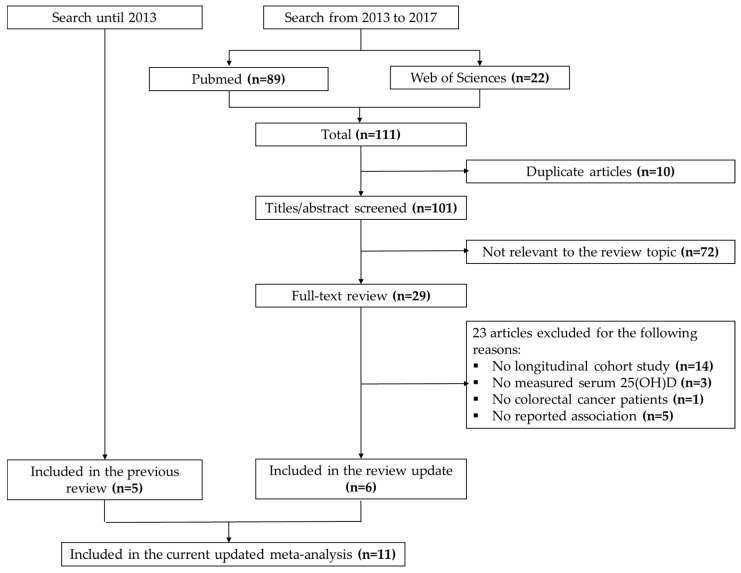
Flow diagram of the systematic literature search for colorectal cancer in PubMed and Web of Science.

A flow diagram illustrating the results of the literature search is shown in Figure 1. First, we included the five studies published prior to 2013, which were included in our previous meta-analysis [10]. In addition to that, the electronic databases search conducted in September 2017 yielded a total of 111 citations published between 2013 and 2017. After excluding duplicates, titles and abstracts of 101 articles were screened, of which 72 articles were excluded as non-relevant. The remaining 29 potentially relevant articles underwent a full-text review, of which six studies were selected eligible for inclusion in our meta-analysis. Subsequently, our current meta-analysis includes 11 eligible studies with a total of 7718 participants. A list of excluded articles is shown in Supplementary Materials—List of excluded studies. Results from two additional studies, not included in the meta-analysis because 25(OH)D levels were predicted rather than measured [19,20] were only considered in a sensitivity analysis.

### 3.2. Study Characteristics

Study characteristics and main results of the eligible studies are provided in Table 1. Four studies were conducted in the United States of America (USA) [5,21,22,23], five studies in Europe (multiple-sites [3], Scotland [24], Norway [25], Germany [26], and Italy [27]), and two in Asia [4,28]. The sample size of the included studies ranged from 52 to 2832 participants. Six studies investigated stage I–IV patients [3,4,5,21,25,26], two studies investigated stage I–III patients [24,28] and three studies investigated exclusively stage IV patients [22,23,27]. Three studies employed no, or only very limited, adjustment for covariates [23,27,28], while the remaining eight studies adjusted for age, sex, season and other clinical characteristics. Three studies measured blood 25(OH)D levels using liquid chromatography mass spectrometry, the gold standard for measuring vitamin D [21,24,26]. The remaining studies [3,4,5,22,23,25,27] measured serum 25(OH)D with radioimmunoassay except one study [28] that used enzyme linked immunoassay. Overall, the highest 25(OH)D category cut-off observed in the included studies ranged between 33 and 188 nmol/L and the lowest 25(OH)D category cut-off ranged between 5 and 75 nmol/L.

### 3.3. Meta-Analysis

**Figure 2:**
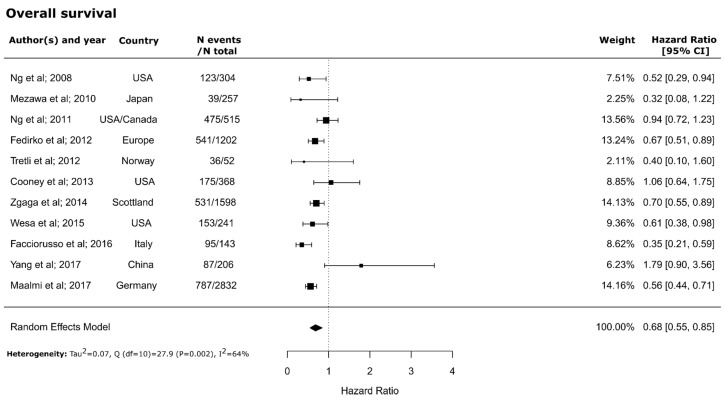
Forest plot for the association of high versus low 25-hydroxyvitamin D_3_ (25(OH)D) levels with overall survival in patients with colorectal cancer. CI: Confidence interval.

**Figure 3:**
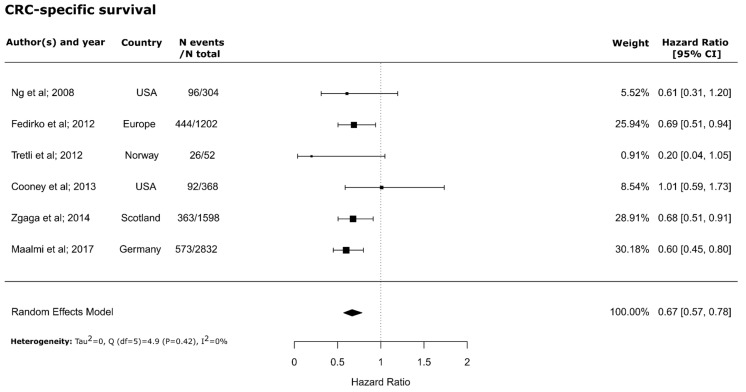
Forest plot for the association of high versus low 25-hydroxyvitamin D_3_ (25(OH)D) levels with CRC-specific survival in patients with colorectal cancer. CI: Confidence interval.

The meta-analysis revealed significantly higher overall (HR = 0.68; 95% CI = 0.55–0.85) and CRC-specific survival (HR = 0.67; 95% CI = 0.57–0.78) in patients with higher blood 25(OH)D concentrations compared to those with lower concentrations. The forest plots of within-study risk estimates are shown in Figure 2 and Figure 3. No significant heterogeneity between the studies was found for CRC-specific survival (Q (*df* = 5) = 4.9, *P*-value = 0.42; I^2^ = 0%). However, a significant but moderate heterogeneity between studies was found for overall survival (Q (*df* = 10) = 27.9, *P*-value = 0.002; I^2^= 64%). No evidence for publication bias was found for either overall (Kendall’s tau = −0.09; *P* = 0.76; Egger’s t value = −0.68; *P* = 0.49) or CRC-specific survival (Kendall’s tau = −0.06; *P* = 1.0; Egger’s t value = −0.65; *P* = 0.51) (Figure S1).

### 3.4. Sensitivity Analyses

Sensitivity analyses were conducted to examine the stability of the estimates for overall survival. The pooled estimates remained stable after the exclusion of studies with either pre-diagnostically assessed 25(OH)D (HR = 0.70; 95% CI = 0.53–0.91, I^2^ = 70%) or very limited adjustment for covariates (HR = 0.69; 95% CI = 0.57–0.84, I^2^ = 49%) but also after the inclusion of two studies with predicted 25(OH)D concentrations (HR = 0.67; 95% CI, 0.54 to 0.83, I^2^ = 65%).

### 3.5. Subgroup Analyses

Results of the subgroup analyses are shown in Table 2. Studies conducted in Europe, with larger sample size, and including stage I–IV patients showed a more prominent association between 25(OH)D concentrations and overall survival and the lowest level of heterogeneity compared to studies conducted in USA/Asia, with smaller sample size or restricted to a specific CRC stage. In other stratified analyses by year of publication and median follow-up time, the results did not show a difference between subgroups.

### 3.6. Dose-Response Meta-Analysis

**Figure 4:**
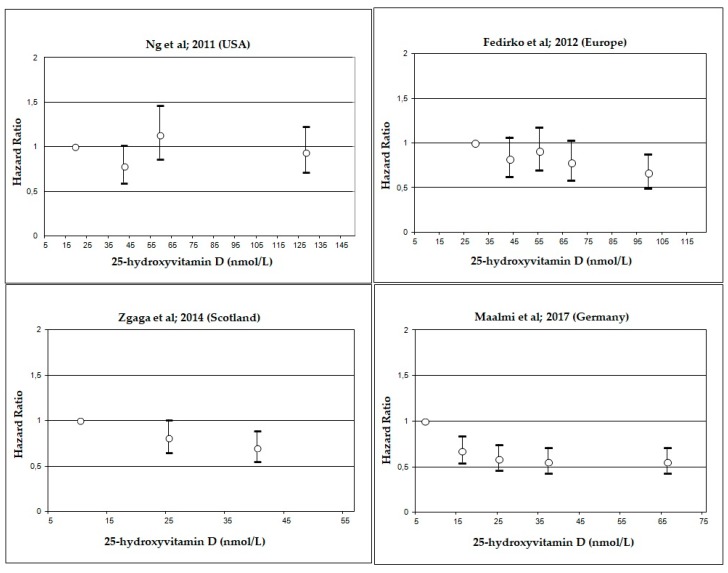
Hazards ratios and 95% confidence intervals for overall survival in colorectal cancer (CRC) patients according to circulating 25-hydroxyvitamin D (25(OH)D) serum concentrations. Depending on available information, medians, mid-points or means of the categories were used for the definition of study specific concentrations of serum 25(OH)D categories.

**Figure 5:**
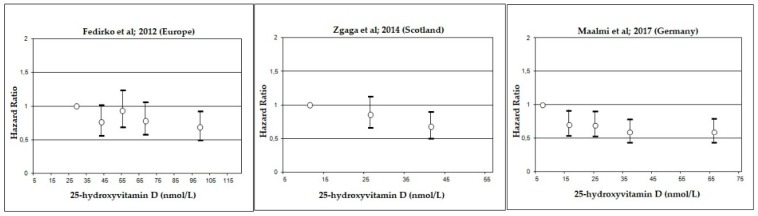
Hazards ratios and 95% confidence intervals for CRC-specific survival in colorectal cancer (CRC) patients according to circulating 25-hydroxyvitamin D (25(OH)D) serum concentrations. Depending on available information, medians, mid-points or means of the categories were used for the definition of study specific concentrations of serum 25(OH)D categories.

Dose-response graphs suggested an improvement in overall (Figure 4) and CRC-specific survival (Figure 5) with 25(OH)D concentrations above 25–50 nmol/L for most studies.

## 4. Discussion

This updated systematic review and meta-analysis found better overall and CRC-specific survival in CRC patients with higher blood 25(OH)D concentrations compared to those with lower concentrations. Associations were most prominent and heterogeneity lowest in studies that were conducted in Europe; had larger sample sizes and included stage I–IV patients.

Compared to our previous meta-analysis which included only 5 studies [10], this updated investigation included 11 studies, which increased the sample size more than 3-fold from 2330 to 7718 participants leading to an increase in the statistical power of our analysis and allowing for subgroup analyses by study and patient characteristics. Moreover, the included studies were conducted in CRC patients in different stages.

Evidence from biological studies supports a role of vitamin D in cancer. Vitamin D up-regulates the transcription of genes involved in the inhibition of proliferation/angiogenesis and genes involved in the inducement of differentiation, apoptosis, and DNA repair mechanisms. Furthermore, several inflammatory processes and the release of cytokines, such as interleukin (IL)-6, IL-8 and IL-17, involved in CRC progression, are regulated by vitamin D [8]. Such a supportive role of Vitamin D in anti-cancer cell mechanisms might be one plausible explanation for the observed associations with CRC prognosis. On the other hand, low 25(OH)D levels may also have a negative influence on survival through causing secondary hyperparathyroidism which has been recently shown to be associated with increased mortality in frail people [29].

Although it is of interest for CRC patients to have high vitamin D levels, the upkeep of these high concentrations is challenging especially for patients undergoing surgery, an invasive procedure that increases the oxidative stress in the body and contributes to a decrease of circulating 25(OH)D concentrations. Moreover, CRC patients undergoing chemotherapy have an additional risk for a drop in circulating 25(OH)D concentration [30]. Even though a causal relationship between vitamin D and survival in CRC patients has not been established yet, the strong and consistent epidemiological evidence supporting it calls for evaluating a potential of monitoring, and eventually supplementing vitamin D to enhance survival and potentially other relevant outcomes such as attenuating chemotherapy side effects [31], fatigue [32] and muscle weakness [33] in randomized controlled trials.

Despite the plausibility of a causal relationship between low vitamin D status and poor survival, the possibility of alternative explanations has also to be kept in mind. In particular, there is an ongoing discussion as to what extent low vitamin D status may simply be an indicator of poor health status rather than a causal factor itself. This concern is especially evident in studies with limited adjustment for covariates related to baseline health status but can never be fully ruled out in observational studies. Evaluating and eventually establishing a causal role in well-designed randomized trials will, therefore, remain indispensable.

Meanwhile, a number of randomized clinical trials (RCTs) investigating the effect of vitamin D supplementation on survival as a primary or secondary outcome among CRC patients have been started. One such intervention study is currently conducted in the USA (ClinicalTrials.gov identifier NCT02603757, study period: 2016–2022), including CRC patients before the start of neoadjuvant or adjuvant chemotherapy and randomizing patients for standard-dose (2000 International Units (IU)/day) versus higher-dose vitamin D (50,000 IU/week). This RCT is aiming to test whether high-dose vitamin D supplementation would rapidly increase vitamin D levels at the time early before and shortly after surgery (but prior to neoadjuvant and adjuvant chemotherapy) and whether vitamin D supplementation could enhance survival. However, this RCT includes CRC patients with baseline 25(OH)D concentrations up to 130 nmol/L. The inclusion of a substantial proportion of CRC patients with sufficient vitamin D levels who might not benefit from vitamin D supplementation may dilute potential intervention effects and compromise study power [34]. On the other hand, the administered doses might not be sufficient for patients with severe vitamin D deficiency.

Two additional RCTs conducted exclusively in CRC patients with metastatic disease have been established. The first RCT (ClinicalTrials.gov identifier NCT01150877, study period: 2011–2017) is being conducted in Canada randomizing patients into standard-dose (2000 IU/day) versus higher-dose vitamin D groups, allowing to raise blood 25(OH)D concentrations to 200–250 nmol/L during a period of 16 months, whereas the second RCT (ClinicalTrials.gov identifier NCT01516216, study period: 2012–2016) is being conducted in the USA, randomizing patients into a 5-fluorouracil, leucovorin and oxaliplatin (FOLFOX) + bevacizumab chemotherapy with standard-dose vitamin D_3_ (400 IU/day) group versus a higher-dose vitamin D (4000 IU/day) group. The results of both trials are not published yet. Due to the very high prevalence of vitamin D deficiency in cancer patients and given the strong and consistent evidence on its association with poor survival from observational studies, correction of vitamin D deficiency might be warranted in these patients even in the absence of results from clinical trials. Since supplementation of up to 10,000 IU per day is recognized as a safe level for all adults [35], such a dose is likely to pose no risk in cancer patients. Even though sunlight exposure can be a cost-effective way to provide cancer patients with the needed amounts of vitamin D [36], this is not likely to be the case for patients who are living in northern latitudes especially in winter. Moreover, CRC patients after surgery are more likely to be homebound and to have limited outdoor physical activity, reducing therefore their sunlight exposure and leaving vitamin D supplementation as a unique alternative to insure providing them with needed amount of vitamin D.

Although RCTs are the gold standard to assess causality of a relationship between exposure and outcome, other new approaches such as the Mendelian randomization-the random assortment of genes from parents to offspring that occurs during gamete formation-provide additional methods to infer causality [37]. In a very large study conducted in Denmark, Afzal et al. [38] found that genetically low 25(OH)D concentrations were causally associated with increased overall and cancer mortality in participants from the general population. Due to the low costs of Mendelian randomization studies compared to RCTs, such a method might offer an interesting complementary option to test for causality between vitamin D status and survival in patients with CRC even though results have to be interpreted with caution due to a potential violation of the underlying assumptions.

If a causal relationship between vitamin D status and CRC prognosis can be corroborated by RCTs, using vitamin D as an adjuvant therapeutic option may be a particularly cost-effective option to enhance prognosis of CRC patients not only in economically affluent countries but also in countries in which resources for high-cost modern therapeutics keep being very limited.

Several limitations of this meta-analysis should be stated. First, significant heterogeneity was observed between the studies for overall mortality but not for CRC-specific mortality. The heterogeneity persisted in most subgroups after stratification and was only significantly reduced among studies conducted in Europe, with larger sample size, and including stage I–IV patients. Some studies included in our meta-analysis did not adjust for some potentially relevant confounders which may have led to residual confounding and may explain some of the observed heterogeneity. Differences in the method used for measuring blood 25(OH)D levels may also be a source of heterogeneity between the included studies. Unfortunately, subgroup analyses for the outcome CRC-specific survival could not be conducted due to the lack of report for the association estimates in many studies. Second, very heterogeneous definitions of vitamin D categories were used across studies. Such variability in exposure levels may likewise explain some of the heterogeneity and limit comparability between studies. Third, in all studies, a single 25(OH)D measurement was conducted for each patient, and the time of blood sampling in relation to diagnosis and therapy varied between studies. Such single measurements may not accurately reflect a patient’ vitamin D status across time. Forth, the exclusion of abstracts from this meta-analysis may have led to missing potentially relevant results not published yet. However, no indication of publication bias was observed.

## 5. Conclusions

The consistent evidence presented across an increasing number of studies, including very large studies, strongly corroborates and expands previously available evidence for better survival of CRC patients with sufficient blood 25(OH)D concentrations. These results, along with the very high prevalence of vitamin D insufficiency among CRC patients [26], suggest a great potential of vitamin D supplementation for enhancing prognosis of CRC patients, which should be thoroughly followed up and evaluated in rigorously designed and conducted RCTs.

## Figures and Tables

**Table 1:** Studies reporting on the association of serum 25(OH)D levels (nmol/L) with overall and CRC-specific mortality among CRC patients.

Author(s) (Year) Study Acronym	Study Population					Association of 25(OH)D with Mortality				Covariates and Stratification Factors
						Overall Survival		CRC-Specific Survival		
	Country (Recruitment Period) FU (Years)	N_total_ (Sex: F/M) (Stage)	All Deaths (CRC Deaths)	Age: Mean/Median (Range)	Time between Diagnosis and Blood Draw/Measurement Method	25(OH)D Levels (nmol/L) Mid-Point/Interval	HR (95% CI)	25(OH)D Levels (nmol/L) Mid-Point/Interval	HR (95% CI)	
**Ng et al. (2008) NHS/HPFS [5]**	United States of America (USA) (1991–2002) Median: 6.5	304 (159/145) (stage I–IV)	123 (96)	68 (All) 43–70 (NHS) 40–75 (HPFS)	**Pre-diagnostic** (>2 years before diagnosis)/RIA	41 59 72 100	1.00 0.81 (0.49–1.35) 0.81 (0.48–1.37) 0.52 (0.29–0.94)	41 59 72 100	1.00 0.76 (0.41–1.42) 1.04 (0.58–1.89) 0.61 (0.31–1.19)	Age, sex, season, BMI, physical activity, race, stage, grade, tumor location, dietary vitamin D intake
**Mezawa et al. (2010) [4]**	Japan (2003–2008) Median: 2.7	257 (92/165)(stage I–IV)	39 (30)	65 (50–80)	**Post-diagnostic** (at operation)/RIA	7.5–17 20–25 28–38 40–90	0.5 (0.16–1.54) 0.55 (0.18–1.65) 1.00 0.16 (0.04–0.63)	Per 2.5 nmol/L	0.98 (0.89–1.08)	Age, sex, season, BMI, physical activity, stage, tumor location, type of resection, number of lymph nodes with metastasis
**Ng et al. (2011) ^a^ N9741 [22]**	USA/Canada (1998–2001) Median: 5.1	515 (209/306)(stage IV)	475 (N.R)	61 (26–85)	**Post-diagnostic** (after chemotherapy)/RIA	5.7–33 33–50 50–68 68–188	1.00 0.78 (0.60–1.02) 1.13 (0.87–1.47) 0.94 (0.72–1.23)	N.R	N.R	Age, sex, race, geographic region, number of metastatic sites, chemotherapy
**Fedirko et al. (2012) EPIC [3]**	Europe (1992–1998) Mean: 6	1202 (606/596) (stage I–IV)	541 (444)	62 (N.R)	**Pre-diagnostic**/EIA	29 43 55 68 99	1.000.82 (0.63–1.07) 0.91 (0.70–1.18) 0.78 (0.59–1.03) 0.67 (0.50–0.88)	29 43 55 68 99	1.000.76 (0.56–1.02) 0.93 (0.69–1.24) 0.78 (0.58–1.06) 0.69 (0.50–0.93)	Age, sex, season, BMI, smoking, physical activity, stage, tumor location, grade, dietary calcium intake
**Tretli et al. (2012) JANUS cohort [25]**	Norway (1973–2007) Range: 0–24	52 (20/32) (stage I–IV)	36 (26)	59 (32–75)	**30 days** (−82; +87) ^b^ /RIA	<46 46–61 62–81 >81	1.00 0.48 (0.18–1.29) 0.61 (0.23–1.59) 0.40 (0.10–1.60)	<44 44–56 56–76 >77	1.00 0.46 (0.15–1.48) 0.73 (0.25–2.15) 0.20 (0.04–1.10)	Age, sex, stage, days between sampling and measurement
**Cooney et al. (2013) [21]**	United States of America (USA) (1994–1998) Mean: 8.03	368 (152/216) (stage I–IV)	175 (92)	64.8 (<85 years)	**Post-diagnostic** (at least 21 days after chemotherapy)/LCMS	<38.7 38.7–52 52.2–61.7 62–77 >77	1.00 1.12 (0.68–1.83) 1.28 (0.78–2.10)1.45 (0.88–2.39)1.06 (0.64–1.75)	<47.5 47.5–66.5 >66.5	1.00 0.96 (0.57–1.63) 1.01 (0.59–1.74)	Age at diagnosis, stage, race, sex, smoking status, month of blood draw, log CRP
**Zgaga et al. (2014) SOCCS cohort [24]**	Scotland (1999–2006) Median: 8.9	1598 (682/916) (stage I–III)	531 (363)	62 (N.R)	**Post-diagnostic** (median of 105 days after surgery)/LCMS	<18 18–33 >33	1.00 0.81 (0.65–1.01) 0.70 (0.55–0.89)	<18 18–33 >33	1.00 0.86 (0.66–1.13) 0.68 (0.50–0.90)	Age, sex, season, stage, tumor site, surgery, time between definitive treatment and sampling, BMI, physical activity
**Wesa et al. (2015) [23]**	United States of America (USA) (2005–2006) Median: 3.4	241 (N.R) (stage IV)	153 (N.R)	63 (52–73)	**Post-diagnostic** (±30 days)/RIA	<75 ≥75	1.00 0.61 (0.38–0.98)	N.R	N.R	Albumin, ECOG performance status
**Facciorusso et al. (2016) [27]**	Italy (1999–2011) Median: 6	143 (41/102) (stage IV)	95 (89)	68 (41–85)	**Post-diagnostic** (after chemotherapy)/RIA	≤50 >50	1.00 0.35 (0.21–0.59)	N.R	N.R	Carcinoembryonic Antigen, number of nodules, nodule size
**Yang et al. (2017) [28]**	China (2011–2012) Median: 3.75	206 (75/131) (stage I–III)	87 (N.R)	63 (30–85)	**Post-diagnostic** (7 days before surgery)/ELISA	<15.5 15.5–74.75 >74.75	1.00 1.18 (0.71–1.94) 1.79 (0.90–3.56)	N.R	N.R	Not adjusted
**Maalmi et al. (2017) DACHS study [26]**	Germany (2003–2010) Median: 4.8	2832 (1178/1732) (stage I–IV)	787 (573)	68 (30–96)	**Post-diagnostic** (36 days)/LCMS	<12 12–20 20–30 30–45 >45	1.00 0.68 (0.55–0.84) 0.59 (0.47–0.74) 0.56 (0.44–0.71) 0.56 (0.44–0.71)	12 12–20 20–30 30–45 >45	1.00 0.71 (0.55–0.92) 0.70 (0.54–0.91) 0.60 (0.45–0.79) 0.60 (0.45–0.80)	Sex, age, season, stage, history of: diabetes, hypertension and cardiovascular diseases, tumor location, tumor detection mode, BMI, surgery, smoking, chemotherapy, physical activity, time between diagnosis and blood draw

FU: Follow-up; 25(OH)D: 25-hydroxyvitamin D; CRC: Colorectal cancer; BMI: Body mass index; HR: Hazard ratio; CI: Confidence interval; RIA: Radio immunoassay; LCMS: Liquid chromatography mass spectrometry; ELISA: enzyme-linked immunosorbent assay; EIA: Enzyme immunoassay; NHS: Nurses’ Health Study; HPFS: Health Professionals Follow-Up Study; N9741: National Intergroup Trial of Chemotherapy For Metastatic Colorectal Cancer; EPIC: European Prospective Investigation into Cancer and Nutrition; JANUS: The Janus Serum Bank Cohort; DACHS: Darmkrebs: Chancen der Verhütung durch Screening; SOCCS: The Study of Colorectal Cancer in Scotland; M: male; F: female; ECOG: Eastern Cooperative Oncology Group performance status; CRP: C-reactive protein; N.R: not reported. ^a^ The authors repeated the analyses with a predicted 25(OH)D score in a larger sample of the same study population. ^b^ Negative values indicate that 25(OH)D was measured before diagnosis and positive values indicate that 25(OH)D was measured after diagnosis.

**Table 2:** Stratification analyses of the association between 25(OH)D concentrations (High vs. low) and overall survival in CRC patients.

Stratification Factor		No. of Studies/Patients	Random-Effects Model HR (95% CI)	Q (*df*)	Heterogeneity, I^2^	Kendall’s Tau	Egger’s Test
Overall		11/8555	0.68 (0.55–0.85)	27.9 (10)	64%	0.76	0.49
Geographic location	Europe	5/5827	0.59 (0.48–0.72)	6.9 (4)	43%	0.81	0.15
	USA/Asia	6/2728	0.82 (0.58–1.16)	12.4 (5)	60%	1.00	0.39
Year	<2013	5/2330	0.68 (0.50–0.92)	7.3 (4)	44%	0.81	0.04
	≥2013	6/6225	0.69 (0.50–0.95)	19.4 (5)	74%	0.46	0.22
Sample size	<1000	8/2923	0.69 (0.47–1.00)	23.5 (7)	70%	0.90	0.30
	≥1000	3/5632	0.63 (0.55–0.73)	1.8 (2)	0%	1.00	0.71
Median	<5 years	4/3536	0.70 (0.42–1.19)	10.7 (3)	72%	0.75	0.82
Follow-time up	≥5 years	7/5019	0.67 (0.53–0.87)	15.7 (6)	62%	0.23	0.20
stage	I–IV	6/5852	0.63 (0.50–0.79)	7.0 (5)	29%	1.00	0.45
	I–III	2/1804	1.05 (0.42–2.63)	6.4 (1)	84%	1.00	1.00
	IV	3/899	0.60 (0.33–1.07)	11.7 (2)	83%	0.33	0.01

25(OH)D: 25-hydroxyvitamin D; CRC: Colorectal cancer; HR: Hazard ratio; CI: Confidence interval; USA: United States of America; *df*: degrees of freedom.

## Supplementary Materials

The following are available online at http://www.mdpi.com/2072-6643/10/7/896/s1, Figure S1: Funnel plots for within-study risk estimates for the highest versus lowest 25-hydroxyvitamin D (25(OH)D) category in relation to overall (A) and CRC-specific survival (B) in colorectal cancer patients. Table S1. MOOSE Checklist. Table S2. Literature search.
