# Supplementary material for: Association between Blood 25-Hydroxyvitamin D Levels and Survival in Colorectal Cancer Patients: An Updated Systematic Review and Meta-Analysis
Source: Nutrients. 2018 Jul 13;10(7):896. doi: 10.3390/nu10070896 (PMC6073902; doi:10.3390/nu10070896)
Supplement: Supplementary file 1 [file nutrients-10-00896-s001.pdf]

Association between Blood 25-hydroxyvitamin D Levels and Survival in Colorectal Cancer Patients: An Updated Systematic Review and Meta-analysis Title

Haifa Maalmi[1, 2,*], Viola Walter [1], Lina Jansen [1], Daniel Boakye [1], Ben Schöttker [1, 2], Michael Hoffmeister [1], Hermann Brenner [1, 2, 3]**Appendices**

2. Materials and Methods

2.1. Search strategy

The reporting of meta-analyses of observational studies in epidemiology (MOOSE) guidelines were followed to perform this systematic review and meta-analysis (14) (Table A1). We carried out a systematic literature search in PubMed and Web of Science databases for articles reporting results of cohort studies conducted in CRC patients and assessing the association between blood 25(OH)D concentrations and overall and CRC-specific survival, using a comprehensive list of search terms (Table A2). The current literature search was restricted to articles published from 2013 until September 2017 with no language restrictions, thereby complementing our previous corresponding literature search of articles published up to 2013 (10).

Table S1. MOOSE Checklist.

**Reporting of background should include**	
Problem definition	To date, a number of reviews and meta-analyzes summarized results from studies that investigated the association between serum 25(OH)D levels and survival in patients with CRC. However, due to the small number of included studies, none of the previous reports have explored this association within subgroups.
Hypothesis statement	A number of much larger studies have since been published especially in patients with CRC stage IV, which may improve the statistical power and increase the precision of estimates and thereby help confirm the evidence about the role of vitamin D in CRC prognosis and help exploring the association within subgroups.
Description of study outcome(s)	cohort studies
Type of exposure or intervention used	Measured 25-hydroxyvitamin D
Type of study designs used	Cohort studies
Study population	Patients with colorectal cancer
**Reporting of search strategy should include**	
Qualifications of searchers (e.g. librarians and investigators)	From each included study, data were independently extracted by two investigators (H.M and V.W) using a standardized data extraction form.
Search strategy, including time period included in the synthesis and keywords	Keywords for literature search are in supplementary B. We included five studies published prior to 2013 which were included in our previous meta-analysis and we restricted the current literature search to articles published from 2013 till September 29, 2017.
Effort to include all available studies, including contact with authors	No language restriction

Databases and registries searched	PubMed and ISI Web of Knowledge (Thomson Scientific Technical Support, New York) databases
Search software used, name and version, including special features used (e.g. explosion)	The endnote X7 software was used throughout the literature search process.
Use of hand searching (e.g. reference lists of obtained articles)	Bibliographies of the retrieved papers (only the included studies) were hand searched for additional references
List of citations located and those excluded, including justification	Details of the literature search process are outlined in the flow chart (Figure 1). The citation list of excluded articles is available in supplementary C.
Method of addressing articles published in languages other than English	We placed no restrictions on language; We were able to obtained all articles potentially eligible for inclusion in English language
Method of handling abstracts and unpublished studies	we excluded conference abstracts due to the insufficient data obtained from them.
Description of any contact with authors	none
**Reporting of methods should include**	
Description of relevance or appropriateness of studies assembled for assessing the hypothesis to be tested	We included articles reporting results of cohort studies conducted in CRC patients and assessing the association between serum 25(OH)D levels with overall and CRC-specific mortality. We excluded studies with ***(i)*** no longitudinal design; ***(ii)*** no CRC patients; ***(iii)*** no measurement of serum 25(OH)D and ***(iv)*** no reported measurement of the association between the exposure and the outcome of interest.
Rationale for the selection and coding of data (e.g. sound clinical principles or convenience)	We used a standardized data extraction form to record study characteristics, information on study population as well as data about the time between serum 25(OH)D measurement and cancer diagnosis and the value of 25(OH)D concentrations presenting the mid-points of the ranges of the reported categories.
Assessment of confounding (e.g., comparability of cases and controls in studies where appropriate)	We conducted 5 subgroup analyses to explore the source of the heterogeneity across the studies
Assessment of study quality, including blinding of quality assessors; stratification or regression on possible predictors of study results	We assumed that studies that didn't adjust for at age, sex, and season as studies with low quality, and we assessed a sensitivity analysis to test the stability of the pooled estimates after their exclusion.
Assessment of heterogeneity	The heterogeneity among the included studies was investigated using I^2 and Q test statistics, with a significant evidence of heterogeneity for $I^2 > 50\%$ or a P value less than 0.05 from the Q-test
Description of statistical methods (e.g. complete description of fixed or random effects models, justification of whether the chosen models account for predictors of study results, dose-response models, or cumulative meta-analysis) in sufficient detail to be replicated	We described the method used for each analysis and the software used.

Provision of appropriate tables and graphics	Flow chart: to show the method of studies identification.<br>Table 1: showing characteristics of included studies<br>Table 2: showing Results of Subgroup analysis<br>Figure 2,3: forest plots of the main two meta-analyses conducted<br>Figure 4: Hazards ratios and 95% confidence intervals for overall survival in patients with colorectal cancer according to circulating 25-hydroxyvitamin D (25(OH)D) serum concentrations.<br>Figure 5: Pooled dose-response relationship between serum 25(OH)D levels and the two outcomes investigated.
**Reporting of results should include**	
Graphic summarizing individual study estimates and overall estimate	Figure 2, Figure 3
Table giving descriptive information for each study included	Table 1
Results of sensitivity testing (e.g. subgroup analysis)	Results section/sensitivity analysis sub-section
Indication of statistical uncertainty of findings	95% CI intervals were presented for all analyses together with I^2 values for the two main meta-analyses
**Reporting of discussion should include**	
Quantitative assessment of bias (e.g., publication bias)	Results of Funnel plot and risk of publication bias is highlighted in the results section and supplementary D.
Justification for exclusion (e.g. exclusion of non–English-language citations)	Reasons for exclusion were reported mainly in the methods section
Assessment of quality of included studies	Study quality was judged based on the adjustment level. We considered age, sex, season and stage as important confounders. Implication of the subgroup analyses related to quality assessment was highlighted
**Reporting of conclusions should include**	
Consideration of alternative explanations for observed results	Evidence from biological studies supports the role of vitamin D in cancer. vitamin D regulates the transcription of genes involved in the Inhibition of proliferation/angiogenesis and the transcription of genes responsible for the inducement of differentiation, apoptosis, and DNA repair mechanisms. Furthermore, several inflammatory processes and cytokines such as interleukin 6 and (IL6), IL8 and IL17 involved in CRC progression are regulated by vitamin D.
Generalization of the conclusions (i.e. appropriate for the data presented and within the domain of the literature review)	If a causal relationship between vitamin D status and CRC prognosis is demonstrated, the objective from using vitamin D as a potential therapeutic option for CRC patients may be related to socio-demographic factors.
Guidelines for future research	Due to the low costs of mendelian randomization studies compared to RCTs, such method is warranted to test causality between vitamin D status and survival in patients with colorectal cancer.
Disclosure of funding source	none

Table S2. Literature search

Steps	Search term	Search string
**1**	Vitamin D	"vitamin d"(MeSH Terms) OR "vitamin d"(All Fields)OR "ergocalciferols"(MeSH Terms) OR "ergocalciferols"(All Fields)
**2**	"25 ohd" or "25 (OH)D" or "25-OH-D"	"25 ohd"(All Fields) OR "25 (OH)D"(All Fields) OR "25-OH-D"(All Fields)
**3**	Calcidiol	"calcifediol"(MeSH Terms) OR "calcifediol"(All Fields) OR "calcidiol"(All Fields)
**4**	Cholecalciferol	"cholecalciferol"(MeSH Terms) OR "cholecalciferol"(All Fields)
**5**	Dihydrotachysterol	"dihydrotachysterol"(MeSH Terms) OR "dihydrotachysterol"(All Fields)
**6**	25-dihydroxyvitamin D2	"25-dihydroxyvitamin D2"(All Fields)
**7**	Hydroxycholecalciferols	"hydroxycholecalciferols"(MeSH Terms) OR "hydroxycholecalciferols"(All Fields)
**8**	"vitamin D2" or "vitamin D3"	"vitamin d2"(All Fields) OR "vitamin d3"(All Fields)
**9**	Calcitriol	"calcitriol"(MeSH Terms) OR "calcitriol"(All Fields)
**10**	"hydroxyvitamin d" or "hydroxyvitamin d2" or "hydroxyvitamin d3"	"hydroxyvitamin d"(All Fields) OR "hydroxyvitamin d2"(All Fields) OR "hydroxyvitamin d3"(All Fields)
**11**	alphacalcidol or alfacalcidol	("1-hydroxycholecalciferol"(Substance Name) OR "1-hydroxycholecalciferol"(All Fields) OR "alphacalcidol"(All Fields)) OR ("1-hydroxycholecalciferol"(Substance Name) OR "1-hydroxycholecalciferol"(All Fields) OR "alfacalcidol"(All Fields))
**12**	Colecalciferol	"cholecalciferol"(MeSH Terms) OR "cholecalciferol"(All Fields) OR "colecalciferol"(All Fields)
**13**	1,25-dihydroxyvitamin D	"1,25-dihydroxyvitamin D"(Substance Name) OR "1,25-dihydroxyvitamin D"(All Fields) OR "1,25 dihydroxyvitamin d"(All Fields)
**14**	"25-hydroxyvitamin D3 1-alpha-hydroxylase"	"25-hydroxyvitamin D3 1-alpha-hydroxylase"(All Fields)
**15**	**1 or 2 or 3 or 4 or 5 or 6 or 7 or 8 or 9 or 10 or 11 or 12 or 13 or 14**	
**16**	Mortality	"mortality"(Subheading) OR "mortality"(All Fields) OR "mortality"(MeSH Terms)
**17**	Death	"death"(MeSH Terms) OR "death"(All Fields)
**18**	Survival	"survival"(MeSH Terms) OR "survival"(All Fields)
**19**	**16 or 17 or 18**	
**20**	Colorectal cancer	"Colorectal Neoplasms"(MeSH Terms) OR "Colorectal cancer"(All Fields)
**21**	Colon cancer	"Colonic Neoplasms"(MeSH Terms) OR "colon cancer"(All Fields)
**22**	Rectal cancer	"Rectal Neoplasms"(MeSH Terms) OR "Rectal cancer"(All Fields)
**23**	**20 or 21 or 22**	
**24**	**15 and 19 and 23**	

List of excluded studies

No longitudinal cohort study

1. Conway, F.J.; McMillan, D.C. Plasma vitamin D concentration and survival in colorectal cancer: potential confounding by the systemic inflammatory response. *J. Clin. Oncol.* **2015**, *33*, 224.
2. Guerrieri-Gonzaga, A; Gandini, S. Vitamin D and overall mortality. *Pigm. Cell Melanoma Res.* **2013**, *26*,16–28.
3. Klampfer, L. Vitamin D and colon cancer. *World J. Gastrointest. Oncol.* **2014**, *6*, 430.
4. Li, M.; Chen, P.; Li, J.; Chu, R.; Xie, D.; Wang, H. Review: the impacts of circulating 25-hydroxyvitamin D levels on cancer patient outcomes: a systematic review and meta-analysis. *J. Clin. Endocrinol. Metab.* **2014**, *99*, 2327–2336.
5. Maalmi, H.; Ordonez-Mena, J.M.; Schottker, B.; Brenner, H. Serum 25-hydroxyvitamin D levels and survival in colorectal and breast cancer patients: systematic review and meta-analysis of prospective cohort studies. *Eur. J. Cancer.* **2014**, *50*, 1510–1521.
6. Mohr, S.B.; Gorham, E.D.; Kim, J.; Hofflich, H.; Cuomo, R.E.; Garland, C.F. Could vitamin D sufficiency improve the survival of colorectal cancer patients? *J. Steroid Biochem. Mol. Biol.* **2015**, *148*, 239–244.
7. Morales-Oyarvide, V; Meyerhardt, J.A.; Ng, K. Vitamin D and Physical Activity in Patients With Colorectal Cancer: Epidemiological Evidence and Therapeutic Implications. *Cancer J.* **2016**, *22*, 223–231.
8. Ng, K. Vitamin D for Prevention and Treatment of Colorectal Cancer: What is the Evidence? *Curr. Colorectal. Cancer Rep.* **2014**, *10*, 339–345.
9. Ou, B.; Zhao, J.; Guan, S; Lu, A. Plasma 25-hydroxyvitamin D levels and survival of colorectal cancer patients: a meta-analysis. *Eur. J. Cancer* **2015**, *51*, 786–788.
10. Pilz, S.; Kienreich, K.; Tomaschitz, A.; Ritz, E.; Lerchbaum, E.; Obermayer-Pietsch, B.; Matzi, V.; Lindenmann, J.; Marz, W.; Gandini, S.; *at al.* Vitamin D and Cancer Mortality: Systematic Review of Prospective Epidemiological Studies. *Anti-Cancer Agents Med. Chem.* **2013**, *13*, 107–117.
11. Shaw, E.; Massaro, N.; Brockton, N.T. The role of vitamin D in hepatic metastases from colorectal cancer. Clin Transl Oncol. *Clin. Translational Oncology* **2017**, *3*, 259–273.
12. Shui, I.; Giovannucci, E. Vitamin D Status and Cancer Incidence and mortality. Adv Exp Med Biol. 2014; 810:33-51.
13. Toriola, A.T.; Nguyen, N.; Scheitler-Ring, K.; Colditz, G.A. Circulating 25-hydroxyvitamin D levels and prognosis among cancer patients: a systematic review. *Cancer Epidemiol Biomarkers Prev.* **2014**, *23*, 917–933.
14. Wang, B.; Jing, Z.; Li, C.; Xu, S.; Wang, Y. Blood 25-hydroxyvitamin D levels and overall mortality in patients with colorectal cancer: a dose-response meta-analysis. *Eur. J. Cancer* **2014**, *50*, 2173–2175.

No measured serum 25(OH)D

1. Fuchs, M.A.; Yuan, C.; Sato, K.; Niedzwiecki, D.; Ye, X.; Saltz, L. B.; Mayer R.J.; Mowat, R.B.; Whittom, R.; Hantel, A.; *at al.* Predicted vitamin D status and colon cancer recurrence and mortality in CALGB 89803 (Alliance). *Ann. Oncol.* **2017**, *28*, 1359–1367.
2. Perna, L.; Hoffmeister, M.; Schöttker, B.; Arndt, V.; Haug, U.; Holleczek, B.; Burwinkelde ,B.; Ordóñez-Menaa, J.M.; Brenner, H. Vitamin D receptor polymorphism and colorectal cancer-specific and all-cause mortality. *Cancer Epidemiol.* **2013**, *37*, 905–907.
3. Yang, B.; McCullough, M.L.; Gapstur, S.M.; Jacobs, E.J.; Bostick, R.M.; Fedirko, V.; Dana Flanders, W.; Campbell, P.T. Calcium, vitamin D, dairy products, and mortality among colorectal cancer survivors: the Cancer Prevention Study-II Nutrition Cohort. *J. Clin. Oncol.* **2014**, *32*, 2335–2343.

No colorectal cancer patients

1. Deng, X.; Song, Y.; Manson, J.E;., Signorello, L.B.; Zhang, S.M.; Shrubsole, M.J.; Ness, R.M.; Seidner, D.L.; Dai, Q. Magnesium, vitamin D status and mortality: results from US National

Health and Nutrition Examination Survey (NHANES) 2001 to 2006 and NHANES III. *BMC Med.* **2013**, *11*, 187.

No reported association

1. Giessen, C.; Nagel, D.; Glas, M.; Spelsberg, F.; Lau-Werner, U.; Modest, D.P.; Michl, M.; Heinemann, V.; Stieber, P.;Schulz, C. Evaluation of preoperative serum markers for individual patient prognosis in stage I-III rectal cancer. *Tumour Biol.* **2014**, *35*, 10237–10248.
2. Giessen-Jung, C.; Nagel, D.; Glas, M.; Spelsberg, F.; Lau-Werner, U.; Modest, D.P.; Schulz, C.; Heinemann, V.; Gioia, D.D.; Stieber, P. Preoperative serum markers for individual patient prognosis in stage I-III colon cancer. *Tumour Biol.* **2015**, *36*, 7897–7906.
3. Obermannova, R.; Dusek, L.; Greplova, K.; Jarkovsky, J.; Sterba, J.; Vyzula, R.; Demlova, R.; Zdrazilova-Dubska, L.; Valik, D. Time-course pattern of blood 25-hydroxycholecalciferol is a significant predictor of survival outcome in metastatic colorectal cancer: a clinical practice-based study. *Neoplasma.* **2015**, *62*, 958–965.
4. Ulrich, C.M.; Toriola, A.T.; Siegel, E.M.; Brenner, H.; Chang-Claude, J.; Abbenhardt, C.; Kotzmann, J.; Song, X.; Owen, R.W.; Hoffmeister, M.; *at al.* Plasma 25-hydroxyvitamin D3, folate and vitamin B12 biomarkers among international colorectal cancer patients: a pilot study. *J. Nutr. Sci.* **2013**, *2*, e9.
5. Winkels, R.M.; Heine-Bröring, R.C.; Van Zutphen, M.; van Harten-Gerritsen, S.; Kok, D.E.; Van Duijnhoven, F.J.; Kampman, E. The COLON study: Colorectal cancer: Longitudinal, Observational study on Nutritional and lifestyle factors that may influence colorectal tumour recurrence, survival and quality of life. *BMC Cancer* **2014**, *14*, 374

3.3. Meta-analysis

The meta-analysis revealed significantly higher overall (HR = 0.68; 95% CI = 0.55–0.85) and CRC-specific survival (HR = 0.67; 95% CI = 0.57–0.78) in patients with higher blood 25(OH)D concentrations compared to those with lower concentrations. The forest plots of within-study risk estimates are shown in Figures 2 and 3. No significant heterogeneity between the studies was found for CRC-specific survival (Q (df = 5) = 4.9, *P*-value = 0.42; I^2 = 0%). However, a significant but moderate heterogeneity between studies was found for overall survival (Q (df = 10) = 27.9, *P*-value = 0.002; I^2= 64%). No evidence for publication bias was found for either overall (Kendall's tau = −0.09; *P* = 0.76; Egger's t value = −0.68; *P* = 0.49) or CRC-specific survival (Kendall's tau = −0.06; *P* = 1.0; Egger's t value = −0.65; *P* = 0.51) (Figure. S1).

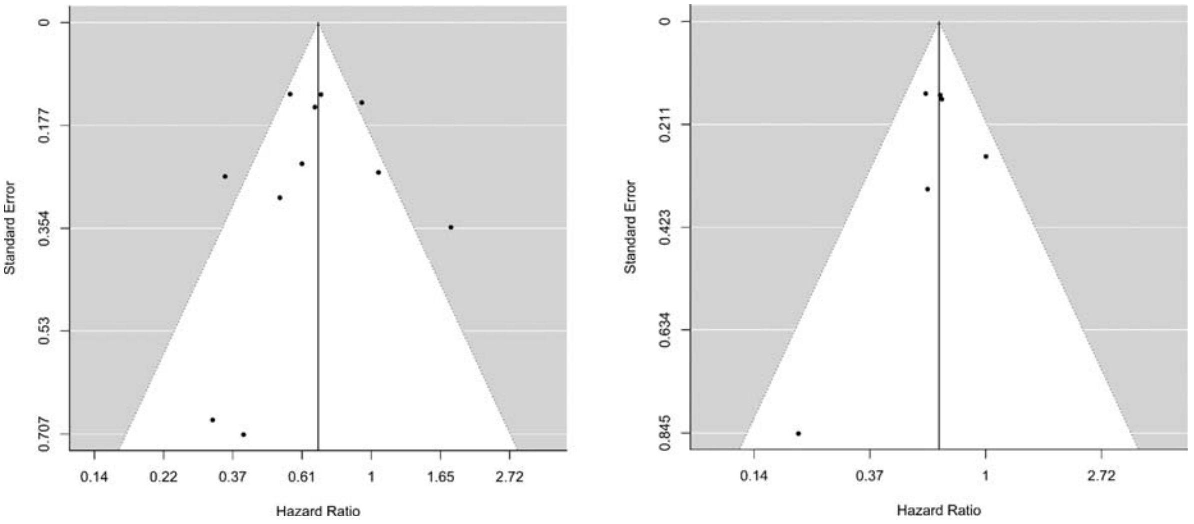

A B

Figure S1. Funnel plots for within-study risk estimates for the highest versus lowest 25-hydroxyvitamin D (25(OH)D) category in relation to overall **(A)** and CRC-specific survival **(B)** in

colorectal cancer patients.

References

1. Conway, F.J.; McMillan, D.C. Plasma vitamin D concentration and survival in colorectal cancer: potential confounding by the systemic inflammatory response. *J. Clin. Oncol.* **2015**, *33*, 224.
2. Guerrieri-Gonzaga, A; Gandini, S. Vitamin D and overall mortality. *Pigm. Cell Melanoma Res.* **2013**, *26*,16–28.
3. Klampfer, L. Vitamin D and colon cancer. *World J. Gastrointest. Oncol.* **2014**, *6*, 430.
4. Li, M.; Chen, P.; Li, J.; Chu, R.; Xie, D.; Wang, H. Review: the impacts of circulating 25-hydroxyvitamin D levels on cancer patient outcomes: a systematic review and meta-analysis. *J. Clin. Endocrinol. Metab.* **2014**, *99*, 2327–2336.
5. Maalmi, H.; Ordonez-Mena, J.M.; Schottker, B.; Brenner, H. Serum 25-hydroxyvitamin D levels and survival in colorectal and breast cancer patients: systematic review and meta-analysis of prospective cohort studies. *Eur. J. Cancer.* **2014**, *50*, 1510–1521.
6. Mohr, S.B.; Gorham, E.D.; Kim, J.; Hofflich, H.; Cuomo, R.E.; Garland, C.F. Could vitamin D sufficiency improve the survival of colorectal cancer patients? *J. Steroid Biochem. Mol. Biol.* **2015**, *148*, 239–244.
7. Morales-Oyarvide, V; Meyerhardt, J.A.; Ng, K. Vitamin D and Physical Activity in Patients With Colorectal Cancer: Epidemiological Evidence and Therapeutic Implications. *Cancer J.* **2016**, *22*, 223–231.
8. Ng, K. Vitamin D for Prevention and Treatment of Colorectal Cancer: What is the Evidence? *Curr. Colorectal. Cancer Rep.* **2014**, *10*, 339–345.
9. Ou, B.; Zhao, J.; Guan, S; Lu, A. Plasma 25-hydroxyvitamin D levels and survival of colorectal cancer patients: a meta-analysis. *Eur. J. Cancer* **2015**, *51*, 786–788.
10. Pilz, S.; Kienreich, K.; Tomaschitz, A.; Ritz, E.; Lerchbaum, E.; Obermayer-Pietsch, B.; Matzi, V.; Lindenmann, J.; Marz, W.; Gandini, S.; *at al.* Vitamin D and Cancer Mortality: Systematic Review of Prospective Epidemiological Studies. *Anti-Cancer Agents Med. Chem.* **2013**, *13*, 107–117.
11. Shaw, E.; Massaro, N.; Brockton, N.T. The role of vitamin D in hepatic metastases from colorectal cancer. Clin Transl Oncol. *Clin. Translational Oncology* **2017**, *3*, 259–273.
12. Shui, I.; Giovannucci, E. Vitamin D Status and Cancer Incidence and mortality. Adv Exp Med Biol. 2014; 810:33-51.
13. Toriola, A.T.; Nguyen, N.; Scheitler-Ring, K.; Colditz, G.A. Circulating 25-hydroxyvitamin D levels and prognosis among cancer patients: a systematic review. *Cancer Epidemiol Biomarkers Prev.* **2014**, *23*, 917–933.
14. Wang, B.; Jing, Z.; Li, C.; Xu, S.; Wang, Y. Blood 25-hydroxyvitamin D levels and overall mortality in patients with colorectal cancer: a dose-response meta-analysis. *Eur. J. Cancer* **2014**, *50*, 2173–2175.
15. Fuchs, M.A.; Yuan, C.; Sato, K.; Niedzwiecki, D.; Ye, X.; Saltz, L. B.; Mayer R.J.; Mowat, R.B.; Whittom, R.; Hantel, A.; *at al.* Predicted vitamin D status and colon cancer recurrence and mortality in CALGB 89803 (Alliance). *Ann. Oncol.* **2017**, *28*, 1359–1367.
16. Perna, L.; Hoffmeister, M.; Schöttker, B.; Arndt, V.; Haug, U.; Holleczek, B.; Burwinkelde ,B.; Ordóñez-Menaa, J.M.; Brenner, H. Vitamin D receptor polymorphism and colorectal cancer-specific and all-cause mortality. *Cancer Epidemiol.* **2013**, *37*, 905–907.
17. Yang, B.; McCullough, M.L.; Gapstur, S.M.; Jacobs, E.J.; Bostick, R.M.; Fedirko, V.; Dana Flanders, W. ; Campbell, P.T. Calcium, vitamin D, dairy products, and mortality among colorectal cancer survivors: the Cancer Prevention Study-II Nutrition Cohort. *J. Clin. Oncol.* **2014**, *32*, 2335–2343.
18. Deng, X.; Song, Y.; Manson, J.E;., Signorello, L.B.; Zhang, S.M.; Shrubsole, M.J.; Ness, R.M.; Seidner, D.L.; Dai, Q. Magnesium, vitamin D status and mortality: results from US National Health and Nutrition Examination Survey (NHANES) 2001 to 2006 and NHANES III. *BMC Med.* **2013**, *11*, 187.
19. Giessen, C.; Nagel, D.; Glas, M.; Spelsberg, F.; Lau-Werner, U.; Modest, D.P.; Michl, M.; Heinemann, V.; Stieber, P.;Schulz, C. Evaluation of preoperative serum markers for individual patient prognosis in stage I-III rectal cancer. *Tumour Biol.* **2014**, *35*, 10237–10248.
20. Giessen-Jung, C.; Nagel, D.; Glas, M.; Spelsberg, F.; Lau-Werner, U.; Modest, D.P.; Schulz, C.; Heinemann, V.; Gioia, D.D.; Stieber, P. Preoperative serum markers for individual patient prognosis in stage I-III colon cancer. *Tumour Biol.* **2015**, *36*, 7897–7906.
21. Obermannova, R.; Dusek, L.; Greplova, K.; Jarkovsky, J.; Sterba, J.; Vyzula, R.; Demlova, R.; Zdrazilova-Dubska, L.; Valik, D. Time-course pattern of blood 25-hydroxycholecalciferol is a significant predictor of

survival outcome in metastatic colorectal cancer: a clinical practice-based study. *Neoplasma.* **2015**, *62*, 958–965.
22. Ulrich, C.M.; Toriola, A.T.; Siegel, E.M.; Brenner, H.; Chang-Claude, J.; Abbenhardt, C.; Kotzmann, J.; Song, X.; Owen, R.W.; Hoffmeister, M.; *at al.* Plasma 25-hydroxyvitamin D3, folate and vitamin B12 biomarkers among international colorectal cancer patients: a pilot study. *J. Nutr. Sci.* **2013**, *2*, e9.
23. Winkels, R.M.; Heine-Bröring, R.C.; Van Zutphen, M.; van Harten-Gerritsen, S.; Kok, D.E.; Van Duijnhoven, F.J.; Kampman, E. The COLON study: Colorectal cancer: Longitudinal, Observational study on Nutritional and lifestyle factors that may influence colorectal tumour recurrence, survival and quality of life. *BMC Cancer* **2014**, *14*, 374.
